# Nutritional interventions in prehabilitation for cancer surgery

**DOI:** 10.1097/MCO.0000000000000974

**Published:** 2023-08-22

**Authors:** Joshua Wall, Melanie Paul, Bethan E. Phillips

**Affiliations:** a Medical Research Council (MRC)/ Versus Arthritis Centre for Musculoskeletal Ageing Research (CMAR) and Nottingham National Institute of Health Research (NIHR) Biomedical Research Centre, School of Medicine, University of Nottingham; b Department of Surgery, Royal Derby Hospital, Derby, UK

**Keywords:** cancer surgery, malnutrition, nutritional supplementation, prehabilitation

## Abstract

**Purpose of review:**

Nutrition remains a key focus in the preoptimization of patients undergoing cancer surgery. Given the catabolic nature of cancer, coupled with the physiological insult of surgery, malnutrition (when assessed) is prevalent in a significant proportion of patients. Therefore, robust research on interventions to attenuate the detrimental impact of this is crucial.

**Recent findings:**

As a unimodal prehabilitation intervention, assessment for malnutrition is the first step, as universal supplementation has not been shown to have a significant impact on outcomes. However, targeted nutritional therapy, whether that is enteral or parenteral, has been shown to improve the nutritional state of patients’ presurgery, potentially reducing the rate of postoperative complications such as nosocomial infections. As part of multimodal prehabilitation, the situation is more nuanced given the difficulty in attribution of effects to the differing components, and vast heterogeneity in intervention and patient profiles.

**Summary:**

Multimodal prehabilitation is proven to improve length of hospital stay and postoperative outcomes, with nutrition forming a significant part of the therapy given. Further work is required to look at not only the interplay between the optimization of nutritional status and other prehabilitation interventions, but also how to best select which patients will achieve significant benefit.

## AN INTRODUCTION TO NUTRITIONAL PREHABILITATION IN CANCER CARE

Cancer prehabilitation is a process on the continuum of care that occurs between the time of cancer diagnosis and the beginning of acute treatment (i.e., surgical resection). This is often based around the identification of impairments, which may be physical and/or psychological, and the subsequent delivery of targeted interventions to improve a patient's ‘resilience’ to treatment. There is a growing body of evidence to support the notion that cancer prehabilitation can reduce both the incidence and severity of current and future clinical insults [1]. Specific to nutritional prehabilitation, patients with cancer often face nutritional challenges, with up to 80% of patients with cancer found to have varying degrees of malnutrition [2^▪▪^], largely attributed to inflammatory mediators released in response to tumor cells [3]. As such, optimization of nutritional status and associated physiological parameters is often a key target of prehabilitation regimes. This review serves to highlight current evidence in the field of nutritional prehabilitation for cancer surgery, both as an independent strategy and as part of multimodal interventions. 

**Box 1:**
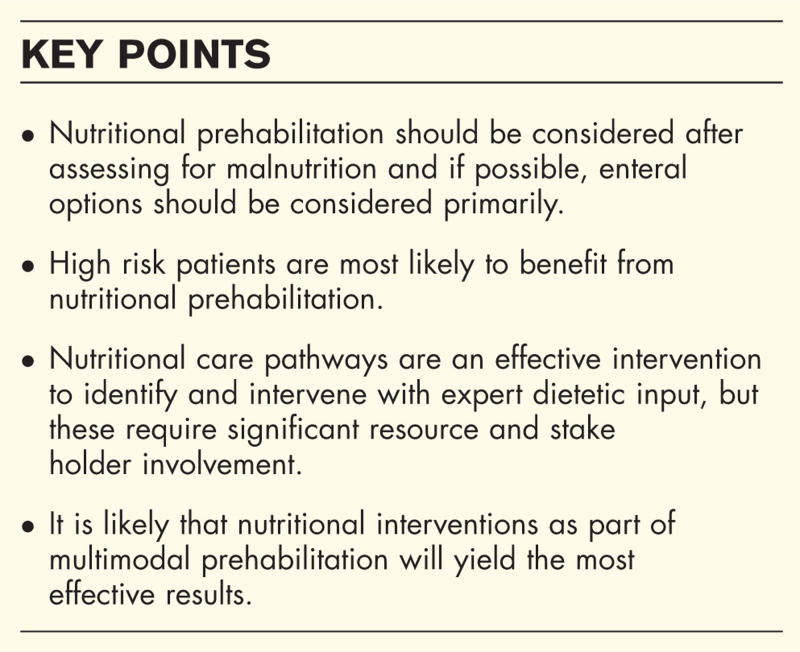
no caption available

## NUTRITIONAL SUPPORT AS INDEPENDENT PREHABILITATION FOR CANCER SURGERY

Cancers such as esophago-gastric and head and neck tumors usually have a more marked and dramatic nutritional impact on patients at the time of presentation due to symptoms such as dysphagia, which coupled with a tendency towards a later staging at diagnosis, can seriously hinder attempts to achieve a curative outcome [4]. In these cancer types in particular, but also in others such as pancreatic cancer or patients undergoing neoadjuvant treatment [5,6], both nutritional screening, subsequent assessment of at-risk patients, then intervention, is key to being able to improve morbidity, mortality and psychological outcomes in patients undergoing oncological resections [7]. In support of this, evidence from Gillis and colleagues used pooled data from five prehabilitation studies to demonstrate that in patients with colorectal cancer, those with malnutrition suffer poor physical and mental health before elective resection [8].

Numerous studies have looked at various methods of screening to identify patients at risk of malnutrition prior to cancer surgery, with a commonly stated aim of facilitating appropriate nutritional intervention. Despite this, a universal recommendation, either within or across cancer types, on how best to screen patients with cancer has not been reached. A recent systematic review by Deftereos *et al.*[9], focusing on gastrointestinal (GI) cancers, suggested that recommendations regarding the use of one screening tool over another could not be made, but that screening tools well validated in general clinical populations, and if possible other oncology populations, should be used.

Despite a relative wealth of literature on nutritional screening and assessment tools, fewer studies have looked at the impact of nutritional interventions alone to attenuate the known risks associated with poor nutritional status such as poor wound healing and infectious complications. Further, the varying effects of different cancers on often heterogeneous cohorts of patients make the formulation of overarching guidelines that apply to all scenarios extremely difficult to achieve. As such, those studies that do exist often focus on the groups of patients in whom malnutrition is most prevalent and/or impactful, such as those highlighted above. However, even in these high-risk groups limited evidence exists. Cantwell *et al.*[10] performed a systematic review of nutritional prehabilitation (excluding parenteral nutrition) in head and neck cancer, and found not only a low yield of eligible studies (*n* = 2) but also no significant improvements in surgical outcomes or physical function.

Oral nutritional supplementation (ONS) is a widely used method to add nutrients in patients at-risk of malnutrition or who are under nourished and not achieving their target dietary intake. However, the impact of its use in the preoperative period and whether it should be used selectively or in all patients with cancer undergoing surgery is still debated. A systematic review by Reece and colleagues exploring the impact of ONS for patients undergoing surgery for GI cancer reported limited evidence for the use of ONS to increase dietary intake or positively influence body weight. They did however conclude that this was likely due to populations, interventions, outcomes and follow-up timeframes each being variable across studies, and that further research into optimal interventions and their timing is needed [11]. Further, with regards to the possibility of preoperative ONS improving postoperative feeding tolerance, He *et al.*[12] randomized 67 patients undergoing sub-total or total gastrectomy to preoperative ONS or dietary advice alone. They found that preoperative ONS did not improve the incidence of GI intolerance, and there was no significant difference in postoperative complication rates between the two groups.

Clearly for any nutritional intervention to have a positive impact, feasibility in terms of delivery and uptake/compliance is key. With regards to the delivery and uptake (i.e., whether patients engage and consume them) of ONS there is evidence to support its ease in delivery to patients. In a single-site randomized trial, Serrano *et al.*[13] conducted a feasibility trial comparing ONS with placebo. Providing 30-days of preoperative ONS which continued for 5 days after surgery, no significant differences in the rate of postoperative complications were reported, although a slightly higher rate of infectious complications in the placebo group was observed. Importantly for a feasibility study, the overall compliance rate was high at 80%. Deftereos *et al.*[14^▪^] underwent a mixed-methods multicenter study to analyze the implementation of a standardized nutrition care pathway in patients with upper GI cancer undergoing surgery. They used a validated theoretical framework for implementation and performed surveys and interviews the staff involved. Patients were highly appreciative of the service, which included a dietetic-led structured and standardized preoperative outpatient service, and dieticians found there to be more proactive engagement with nutritional support. However, the main barriers were the need for significant funding to enable a dietician-led care pathway as well as integrating the service to the existing pathways, suggestive that a ‘one-size fits all’ approach may not be optimal in creating a successful nutritional prehabilitation service.

One emerging trial which may be able to determine whether ONS does impact postoperative complications is the PeriNutri trial. A prospective, multicenter randomized controlled trial, PeriNutri will explore if perioperative ONS in patients with colon cancer undergoing resectional surgery decreases the rate of postoperative 30-day morbidity, and also includes a 5-year follow-up for disease-free and overall survival [15]. This trial offers the possibility of a high-quality study to explore the impact of ONS on both short- and long-term postoperative morbidity in patients with colorectal cancer, although the conclusion may need to be slightly more nuanced when adjusting for other factors such as immediate postoperative complications (i.e., ileus) that may impact the ability of patients to adequately absorb ONS in the inpatient period. Further, given the gold-standard for colorectal surgery management in the elective setting is enhanced recovery after surgery (ERAS)-based (which is multimodal), it may still be difficult to tease out the specific effects elicited by nutritional interventions.

The use of preoperative parenteral nutritional support (PNS) is less well studied than ONS, with most studies tending to look at PNS in the immediate postoperative in-hospital period [16,17^▪▪^]. This may be due to the enhanced logistical burden associated with its management, as well the higher risk profile associated with PNS such as line infections and displacement. Huang *et al.,* found that although the postsurgical complication rate did not improve after giving PNS to 204 sarcopenic patients undergoing radical gastrectomy (although notably hospital costs did), it did reduce the rate of intra-abdominal infections (PNS vs. control, 5.4% vs. 1.2%, *P* = 0.032) [18]. However, given the increased financial costs associated with PNS as well as a lack of overwhelming evidence for its superiority compared to ONS, it is often used in select cases only.

## NUTRITION AS PART OF MULTIMODAL PREHABILITATION

Time from cancer diagnosis to treatment is understandably expeditious to minimize disease progression. For example, in the UK, the National Cancer Action Team stipulate that first treatment must take place within 31 days of decision to treat [19], and as such any preoperative intervention must be effective within this time window. Considering this time constraint, much research has focused on multimodal prehabilitation, commonly including a nutritional component, with a view to implementing as much positive change in a short as time as possible. However, this approach has led to a wide variety of interventions, outcome measures and, on a global level, uncertainty as to the most promising forms of prehabilitation. Further, little insight into the underpinning mechanisms of adaptation in response to these interventions has been garnered to date.

To illustrate the magnitude of heterogeneity in multimodal prehabilitation it is worth considering the differing modalities employed. Most regimes include some aspect of exercise or physical activity (of varying frequency, intensity, time and type) and nutritional supplementation/advice [1,20], delivered both with and without further interventions. These additional components include, for example, nurse-led phone support [21], psychological support [22–29], motivational support [30], alcohol (reduction) and smoking (cessation) interventions [23,30], ‘medical optimization’ [23,28,31,32], and relaxation techniques [33].

Even when considering just the nutritional components of these interventions, there remains a vast gulf in the degree to which patients are prehabilitated, with many studies offering only advice [21–23,29,34,35] whilst others provide routine supplementation [1,25,30–33]. Beyond this, Bojesen *et al.*[36], although arguably not undertaking prehabilitation in its purest sense, offered nutritional screening alone, leaving management of the screening results up to the clinical care team. This study reported an absolute risk reduction in a complicated postoperative course by over 10%. Conversely, on the other end of the spectrum, Lopes *et al.*[28] took complete control of patient's dietary intake and supplied all nutritional intake to participants. This led to improvements in functional outcomes such as handgrip strength and exercise capacity. To further illustrate this point of intervention variance, a comprehensive systematic review of prehabilitation in patients with esophageal cancer included five studies that included nutritional intervention (versus exercise alone). Again, this ranged from nutritional advice only to varying amounts of differing supplementation (prescribed protein supplementation and direct supplementation with leucine metabolite β-hydroxy-β-methylbutyrate (HMB))[2^▪▪^]. Although the conclusion of this review was that most prehabilitation programs confer at least some benefit in terms of improved physical performance, nutritional state and quality of life, clearly this widely varying research base acts as a barrier to implementation of a universal prehabilitation nutritional intervention. This concern, even in homogeneous groups of cancers, is an opinion eloquently summarized by Heil *et al.*[37^▪^], who concluded that conflicting evidence and complex logistical issues were perceived as significant barriers to implementing prehabilitation in colorectal cancer surgery.

Another consideration regarding intervention heterogeneity is group selection. Naturally, prehabilitation is undertaken in diseased cohorts, but as outlined earlier in this article, many studies focus specifically on high(er)-risk patients [23,31,32,36] or patients with more advanced cancers [24,25]. Results in these groups are promising, and concentrating health resources in specific groups of patients may lead to improved outcomes on both an individual and systematic level. However, it may be questioned if this research focus limits the knowledge base around patients who are at lower risk of poor outcomes. Healthier patients and those with less advanced cancers are arguably more likely to return to their precancer status after the insult of cancer and its treatment, so perhaps optimizing these patients prior to surgical intervention would not reduce mortality and significant morbidity but may well lead to a faster return to ‘normality’ and relatively increased quality-of-life.

Despite this proposition, the concept of high-risk individuals being most responsive to nutritional screening, assessment and/or prehabilitation is well illustrated by Bojesen *et al.*[36], who assessed patients for anemia, low functional capacity and nutritional status. Postassessment and based only on the assumption of action taken on these results (i.e. referral for nutritional counseling and supplementation), patients could expect an absolute risk reduction of a complicated postoperative course of almost 11%, suggesting that risk-screening alone could improve postoperative outcomes. It may however also be that the nutritional status of patients with esophageal cancer (as studied by Bojesen *et al.*) is considerably worse than patients with colorectal cancer for instance, due to difficulties in, for example, swallowing solid foods, and that nutritional prehabilitation that addresses this (i.e., ONS) is more effective in those most affected before intervention [21,38].

**FIGURE 1:**
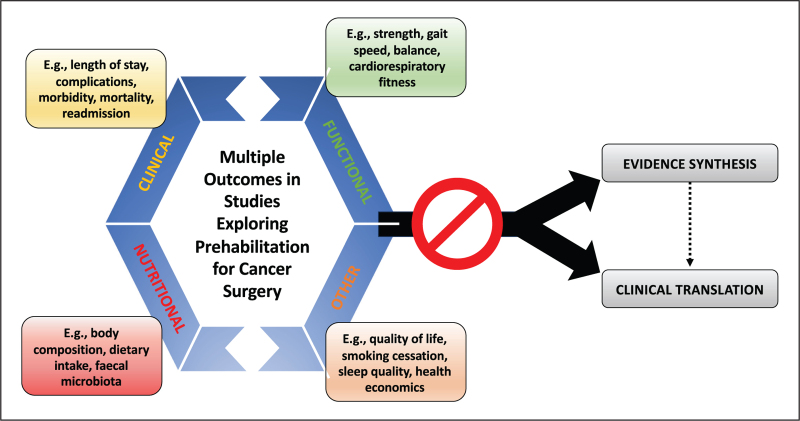
Schematic representation of how the heterogeneity of outcomes in studies exploring nutritional prehabilitation for cancer surgery limit data synthesis and clinical translation.

Beyond a lack of consistency in interventions and patients with a wide variety of cancer being studied (Table 1), the pursuit of optimizing prehabilitation strategies in patients with cancer is further hampered by the plethora of outcome measures. Ignoring feasibility and pilot outcomes, most dependent variables studied can be divided into clinical, functional, nutritional and ‘other’ outcomes (Fig. 1). Clinical outcomes commonly include length-of-stay (LOS) [1,22,25,30–32,35,39], complications/morbidity [1,22,23,25,31,32,36,39], mortality [31,32,39], readmission [1,22,30] and unplanned ITU admissions [36]. Functional outcomes include walking parameters [25,32,35,40], strength [21,31,32,35], and cardiopulmonary exercise test (CPET)-derived parameters [31]. Nutritional outcomes include body composition [21,22,32] and nutritional-intake information [2^▪▪^,^25^,^35^], whilst ‘other’ measures include quality-of-life (QoL) [2^▪▪^,^22^,^25^,^32^], psychological-associated outcomes [22,35], habit cessation [22,25], sleep quality [22,35] and financial cost of care [32,35]. As with the employed interventions, these broad categories veil a large spread of measures. Focusing on the specifics of nutritional outcomes, these are most commonly assessed by a multitude of body composition parameters [2^▪▪^,^21^,^22^,^25^,^32^], dietary habits [22], fecal microbiota [22], and pre/albumin levels [35]- again providing an evidence-base which is challenging to synthesize and translate to recommendations.

**Table 1:** Original research articles supporting this review including key highlights as denoted by bullets

Ref	Author & year	Study design	Cohort & patient number	Intervention	Key finding(s)
[1]	Wooten *et al.*, 2022	Cohort	Abdominal cancer: *n* = 92: Control *n* = 71 Intervention *n* = 21	Home-based 4-week exercise (blood flow restriction) & nutrition (supplement) prehabilitation	Prehabilitation associated with: • Shorter LOS • Decreased incidence of complications • Increased steps on POD 5
[4]	Brookes, 1984	Prospective, observational	Primary squamous cell carcinoma of the head and neck *n* = 114	N/A	- Nutritional deficit was associated with neoplasms of the upper GI tract in >80% of patients - 2-year survival higher in adequately (57.5%) vs. undernourished (7.5%) patients
[5]	Karami *et al.*, 2021	Cross-sectional, descriptive	Patients with cancer, undergoing chemotherapy *n* = 71	N/A	Malnutrition associated with: • age >65 y • male sex • GI cancers
[6]	Wong *et al.*, 2023	Prospective, cohort	Pancreatic adenocarcinoma: *n* = 97: *n* = 72 requested dietician referral, *n* = 31 attended appointment	Patients screened for malnutrition and offered referrals to oncology dietician	- <1/3 of those eligible for referral to an oncology dietician met with one - Reasons for nonattendance included being unable to contact the patient, transfer of care and out-of-pocket fees
[7]	Lorenzon *et al.*, 2020	Survey	Completed by surgeons *n* = 377 participants	N/A	- The use of [nutritional] screening tools is largely neglected - Nutrition is not consistently modified according to risk factors
[8]	Gillis *et al.*, 2021	Pooled-analysis, observational	Colorectal cancer, surgical patients *n* = 266	N/A	- 6MWT was worse with higher patient generated subjective global assessment scores indicating that malnourished patients suffer worse QoL
[12]	He *et al.*, 2022	Prospective, single-blind, randomized controlled trial	Gastric cancer, patients undergoing sub/total gastrectomy: *n* = 67: *n* = 35 advice only *n* = 32 ONS	Preoperative ONS for 7 days prior to surgery or dietary advice-alone	- No difference in postoperative serum indices, prognosis or complications
[13]	Serrano *et al.*, 2022	Randomized, placebo-controlled feasibility trial	GI cancer patients undergoing surgery *n* = 495 screened *n* = 144 eligible n = 71 consented to participation	Protein supplementation for 30 days preoperatively & 5 days postoperatively plus CHO loading on day of operation	- <50% of those eligible agreed to the intervention, mainly due to ‘too much burden’ - 80% median overall compliance
[14^▪^]	Deftereos *et al.*, 2023	Pilot study, controlled trial	Upper GI cancer patients planned for curative surgery: *n* = 70: *n* = 35 control (historical) *n* = 35 intervention	Implementation of a nutritional care pathway	- Implementation of the pathway led to more patients receiving preoperative dietetic intervention
[16]	López-Rodríguez-Arias *et al.*, 2021	Randomized controlled trial	Colorectal cancer patients with normal nutrition, in the immediate postoperative period. Groups further divided by body composition: *n* = 156: *n* = 74 control *n* = 82 intervention	Early peripheral parenteral nutrition	- Early peripheral parenteral nutrition led to a 15.4% reduction in postoperative complications in high-risk vs 1.7% in low-risk body composition - High-risk body composition was associated with increased postoperative complications and LOS - Measuring body composition can identify patients who may benefit from early peripheral parenteral nutrition
[17^▪▪^]	Gao *et al.*, 2022	Randomized clinical trial	Major abdominal surgery in the postoperative period: *n* = 230: *n* = 115 early *n* = 115 late	Early (until POD 3) or late (until POD 8) supplemental parenteral nutrition	Early parenteral nutritional supplementation led to: • Increased energy intake between POD 3–POD 7 • Fewer nosocomial infections • Reduction in antibiotic use
[18]	Huang *et al.*, 2022	Retrospective cohort	Gastric cancer with sarcopenia *n* = 332 (166 matched pairs)	Short-term preoperative parenteral nutrition	Short term parenteral nutrition was: • Not associated with a reduction in the rate of overall complications • Associated with lower intra-abdominal infection rates • Associated with higher hospitalization costs • Advantageous in those with low albumin levels
[20]	Tweed *et al.*, 2021	Feasibility	Colorectal cancer patients aged ≥65 *n* = 30 approached *n* = 9 consented to intervention	4-week multimodal prehabilitation: supervised exercise, prepared protein-rich meals. No control	- 2/3^rd^ of those eligible declined participation - Attendance at ≥80% of exercise sessions was achieved by >75% - 2/3^rd^ accomplished ≥70% compliance with the nutritional program
[21]	Suen *et al.*, 2022	Feasibility	Colorectal cancer patients scheduled for elective surgery *n* = 34 approached *n* = 22 consented to intervention *n* = 2 excluded	2–4 week prehabilitation: supervised exercise, nurse-led phone support and written nutritional information	- Participants attended 79% of exercise sessions and 66% of nurse support calls - Self-reported increase in unsupervised exercise - Increased 6MWT and 30s STS repetitions - Nutritional status and body composition remained unchanged
[23]	de Klerk *et al.*, 2021	Observational retrospective cohort	Colorectal cancer patients scheduled for elective surgery *n* = 351: *n* = 275 control, *n* = 76 intervention	4-week multimodal prehabilitation: personalized exercise, nutritional guidance and treatment of intoxications, polypharmacy and anemia	Prehabilitation was associated with: • Lower rate of complications • Fewer unplanned readmissions • A shorter LOS
[25]	Diaz-Feijoo *et al.*, 2022	Feasibility, controlled	Advanced ovarian cancer patients undergoing cytoreductive surgery *n* = 34: *n* = 19 control (historical) *n* = 15 intervention	Prehabilitation with supervised exercise, nutritional optimization (nutritional counseling & supplementation), and psychological preparation	- Overall adherence to exercise training: 86.7% - Adherence to nutritional optimization: 100% - Adherence to psychological preparation: 80% Prehabilitation associated with: • Shorter LOS • No difference in complications • Shorter time to starting chemo
[26]	Li *et al.*, 2013	Pilot, controlled	Colorectal cancer patients scheduled for elective surgery: *n* = 87: *n* = 45 control *n* = 42 intervention	∼1-week of prehabilitation: home-based exercise (aerobic and RET), nutrition (evaluation by a nutritionist and protein supplementation), and anxiety reduction (90-min with a psychologist & resource for home practice).	- Prehabilitation was associated with improved postoperative functional recovery (functional walking capacity) - No difference in postoperative complications or LOS
[27]	Boukili *et al.*, 2022	Pilot	Patients undergoing major abdominal surgery *n* = 60	4-week trimodal prehabilitation: physical therapy, nutritional support (+/- supplementation) and psychological preparation	- 6MWT, anxiety, depression and QoL items improved after prehabilitation
[29]	Waller *et al.*, 2022	Randomized controlled pilot study	Major abdominal cancer surgery patients: *n* = 22: *n* = 11 control *n* = 11 intervention	Prehabilitation: home-based exercise and nutritional, dietary and meditative advice delivered using a wrist-worn smartwatch connected to a smartphone application	Prehabilitation was associated with: • More daily minutes of physical activity • Greater improvements in 6MWT - No difference in HADS
[31]	Bojesen *et al.*, 2022	Feasibility	Colorectal cancer patients scheduled for elective surgery *n* = 9 eligible *n* = 8 included *n* = 7 completed intervention	Physical training (HIIT & RET) 3 times a week for 4 weeks, nutritional support with supplements and a consultation with a dietician and medical optimization prior to surgery	- Compliance with nutritional support was 57% with half the patients feeling somewhat overwhelmed by the multiple appointments - Most (6/7) reported difficulties with protein supplementation.
[32]	Koh *et al.*, 2022	Nonrandomized, controlled, sequential prospective cohort	Colorectal cancer for curative resection Age ≥70 *n* = 81 *n* = 23 control *n* = 58 intervention	2–4 week prehabilitation program including geriatric assessment, nutritional supplementation and RET	- No improvement in anthropometric or functional characteristics - Shorter LOS in prehab group
[33]	López-Rodríguez-Arias., 2021	Prospective randomized controlled trial	Colorectal cancer patients scheduled for elective surgery *n* = 20: *n* = 10 intervention *n* = 10 control	30-day recommendations for home-based physical exercise, nutritional supplementation and relaxation exercises.	Prehabilitation was associated with: • No significant change in LOS, postoperative complications or loss of lean mass.
[34]	Chen *et al.*, 2017	Randomized controlled trial	Colorectal cancer patients scheduled for elective surgery *n* = 116: *n* = 59 control *n* = 57 intervention	4 weeks of prehabilitation with: aerobic and RET 3 times a week; dietician evaluation and protein supplementation and; relaxation and breathing exercises taught by a psychologist.	Prehabilitation was associated with: • Increased amounts of moderate and vigorous intensity physical activity • Greater improvement of 6MWT
[35]	Li *et al.*, 2022	Retrospective, controlled	Colorectal & gastric cancer patients scheduled for elective surgery *n* = 878: *n* = 439 control *n* = 439 intervention	Sports, nutritional (risk screening and protein intake recommendations) and psychological intervention to finish 7–10 days preop	Prehabilitation was associated with: A better nutritional, sleep quality and psychological and physical function status on preop day 1 • Shorter LOS and lower hospital expenses • Higher QoL at 3 months postop
[36]	Bojesen *et al.*, 2022	Controlled before-and-after study	Colorectal cancer patients scheduled for elective surgery *n* = 1591 *n* = 386 (control hospital, pre intervention period) *n* = 366 (control hospital, postintervention period) *n* = 475 (intervention hospital, pre intervention period) *n* = 364 (intervention hospital, postintervention period)	Screening for anemia, low functional capacity and nutritional status for a minimum of 4 weeks prior to surgery. Surgeon to decide on intervention based on screening results.	- The intervention was associated with a 10.9% absolute risk reduction of a complicated postoperative course, primarily due to a reduction in severe complications.
[37^▪^]	Heil *et al.*, 2022	Qualitative - semi-structured interviews	Healthcare professionals involved in prehabilitation for patients with colorectal cancer *n* = 13	N/A	- Barriers to implementing prehabilitation include the conflicting evidence base on cost-effectiveness and the complex logistical organization - Facilitators included program coordinators, physician leadership and involving skeptical colleagues from the start
[38]	Lidoriki *et al.*, 2022	Observational	Esophago-gastric cancer patients who underwent esophagectomy *n* = 70	N/A	- Lower albumin and geriatric nutritional risk index levels were associated with major complications - Major complications were associated with higher % weight loss, low handgrip strength - Albumin and low muscle mass were associated with anastomotic leakage
[42]	Wang *et al.*, 2023	Observational	GI cancer patients scheduled for elective surgery *n* = 1513	N/A	- 72.8% of the cohort were at risk for malnutrition, this was worse amongst geriatric (age ≥ 65) group - Geriatric patients have decreased muscle mass and skeletal muscle density and more frequently experienced significant weight loss and loss of skeletal muscle - Age ≥65, albumin levels declined functional status and systemic inflammation were independent predictors of postoperative complications

LOS, length of stay; POD, postoperative day.

Inclusion of some outcomes, particularly clinical, may be explained by availability bias; for example, mortality is rarely, if at all, significantly impacted yet is easy to measure [1,2^▪▪^,^20^,^29^,^38^,^39^,^41^] However, the inclusion of such diverse outcomes is concerning, especially given the predominant lack of multivariate analysis and correction for multiple comparisons, not to mention the lack of a clear clinical impact of many of the selected measures. This effect is further confounded when adjusting for the multiple and divergent time-points at which these outcomes are measured [20,23,31,42].

## CONCLUSION

In summary, recent studies investigating both nutritional only and multimodal prehabilitation for cancer surgery appear as a mosaic of interventions and outcomes which serves as a clear limitation to clinical translation. This is reflected in the American Society of Clinical Oncology (ACSO) guidelines which recommends exercise during cancer treatment, but suggests more research is required for nutritional interventions [2^▪▪^]. However, stepping back from the minutia of individual studies reveals a somewhat clearer picture. Multimodal prehabilitation consistently improves LOS [1,25,32,35,39] and QoL [2^▪▪^,^32^], and reduces both complication burden and healthcare associated costs [1,23,25,36]. Importantly given the patient groups in focus, this may lead to an earlier time to adjuvant chemotherapy and/or return to everyday living, and more time spent with loved ones.

Based on existent literature, future work could further investigate the financial cost of prehabilitation versus the cost of LOS, the use of wearable devices to aid prehabilitation such as suggested by Waller *et al.*[29], and/or the involvement of partners in a prehabilitation as suggested by Paterson *et al.*[43]. A final and interesting consideration for future research for nutritional prehabilitation is the interaction between nutrition and exercise during multimodal prehabilitation; specifically, as suggested by Gillis *et al.*[44], does optimized nutrition allow an individual to gain the most from a given exercise regime?

## Acknowledgements

*There are no additional acknowledgements to be made for this article.*

### Financial support and sponsorship

*This work was supported by University Hospitals Derby and Burton NHS Foundation Trust, the Medical Research Council (MRC) (grant number MR/P021220/1) as part of the MRC-Versus Arthritis Centre for Musculoskeletal Ageing Research awarded to the Universities of Nottingham and Birmingham, the MRC (grant number MR/X005240/1) and the NIHR Nottingham Biomedical Research Centre.*

### Conflicts of interest

*There are no conflicts of interest.*

## REFERENCES AND RECOMMENDED READING

Papers of particular interest, published within the annual period of review, have been highlighted as:

▪ of special interest
▪▪ of outstanding interest
